# Effects of Adherence to Statin Therapy on Health Care Outcomes and Utilizations in Taiwan: A Population-Based Study

**DOI:** 10.1155/2015/149573

**Published:** 2015-10-11

**Authors:** Ying-Chun Li, Wei-Ling Huang

**Affiliations:** Institute of Health Care Management, National Sun Yat-sen University, 70 Lien-Hai Road, Kaohsiung 804, Taiwan

## Abstract

*Aim*. Good medication adherence may decrease the probability of worse outcomes and reduce unnecessary medical care costs. This study aims to evaluate medication adherence for people on statin therapy. *Methods*. National health insurance databases were analyzed from January 1, 2001, to December 31, 2007. Study samples were patients of 45 years and older adults who took statin for the first time during the study period. Medication possession ratio (MPR) was measured until the patients had hospitalization or reached the three-year follow-up period. We identified a good (MPR ≥ 80%) and a poor (MPR < 80%) medication adherence group to conduct statistical analyses. *Results.* 40.8% of patients were of good medication adherence and 59.2% were of poor medication adherence. Multivariate logistic regression model indicated that the MPR ≥ 80% group had significantly less probability of hospitalization (*P* < 0.001). Being men, increasing age, higher Charlson Comorbidity Index (CCI) scores, seeking care mostly in the medical center or teaching hospitals, and living in the suburban or rural areas had higher probability of hospitalization (*P* < 0.05 or *P* < 0.001). The MPR ≥ 80% group spent less hospitalization expenditures (*P* < 0.001). *Conclusion.* Effective interventions may be applied to the poor medication adherence group in order to improve their health care outcomes.

## 1. Introduction

Medication treatment is an effective therapy for people with chronic diseases [1–4]. In general, such medication taking involves long term activity. However, whether people with chronic conditions have good medication adherence or not is an important research question. Good medication adherence may reduce unnecessary medical care costs and decrease the probability of bad outcomes [5–7]. A study indicated that the therapeutic effect of a drug depends not only on patients having the treatment prescribed but also on their adherence to or compliance with the treatment [8]. Moreover, does drug treatment reduce overall health care costs by reducing patients' need for expensive medical services such as hospitalization and emergency room (ER) treatment [3]? It is also critical to look at this issue in detail. Researchers suggested that feasible mechanisms of surveillance to monitor and evaluate impact of medication adherence are needed [9]. Meanwhile, what major factors affect individual medication adherence behavior among people with chronic conditions is an essential research and clinical question to be investigated. Effective interventions can be designed based on the study results [10, 11]. All of these issues are critical policy and research topics for the health care delivery system. Nevertheless, researches of such topics are still limited in Taiwan. There is an urgent need to apply system-wide reviews and empirical assessments of these important issues. This research aims to fill some of those research gaps by conducting evaluations of medication adherence for people with statin therapy.

A study indicated that lowering 10% of total cholesterol concentrations may reduce 25% of the probability for having coronary artery disease (CAD) [12]. To decrease cardiovascular disease occurrence, apart from changing diet or life style, statins (3-hydroxy-3-methylglutaryl coenzyme A reductase inhibitors) usually are applied to lower blood cholesterol level and prevent coronary artery disease reoccurrence [13]. Based on the US National Cholesterol Education Program Adult Treatment Panel III, statin had become the first choice medication to control lipid and prevent cardiovascular disease since it can effectively control LDL [14]. A retrospective cohort study in Asia indicated that 66% patients with coronary artery disease can reach effective outcomes after taking statin for three months [15]. Due to the fact that there is an increasing number of patients with chronic cardiovascular disease and statin medication is more expensive than other lowering cholesterol medications, the statin consumptions and related expenditures are also increasing over time in Taiwan. Based on the report from Bureau of National Health Insurance, atorvastatin ranks number two in the medication use and costs NT$1.7 billion with 4.6% growth rate. Rosuvastatin and simvastatin rank numbers 8 and 13 and cost NT$1.2 billion and 0.9 billion, respectively. Rosuvastatin even had 21.6% growth rate. This becomes heavy burdens of the health care delivery system. Therefore, this study emphasized the adherence to statin therapy and proposed to explore (1) associations between medication adherence and medical costs and outcomes and (2) major factors influencing individual medication adherence behaviors.

## 2. Methods

This was a retrospective cohort study. The study period was from January 1, 2001, to December 31, 2007. National health insurance databases were used for analyses. Taiwan launched a single-payer National Health Insurance program on March 1, 1995. As of 2014, 99.9% of Taiwan's population were enrolled. The database of this program contains registration files and original claim data for reimbursement. Large computerized databases derived from this system by the National Health Insurance Administration, Ministry of Health and Welfare, Taiwan, and maintained by the National Health Research Institutes, Taiwan, are provided to scientists in Taiwan for research purposes. This study used data of LHID 2005 which contains all the original claim data of 1,000,000 beneficiaries, where registration data of everyone who was a beneficiary of the National Health Insurance program during the period of January 1, 2005, to January 1, 2006, were drawn for random sampling. There are approximately 25.68 million individuals in this registry. All the registration and claim data of these 1,000,000 individuals collected by the National Health Insurance program constitute the LHID 2005. There was no significant difference in the gender distribution between the patients in the LHID 2005 and the original National Health Insurance Research Database [16].

We focused the study sample on patients 45 years of age and older adults who took statin medication the first time during the study period. This study analyzed five major statin medications including lovastatin, pravastatin, simvastatin, fluvastatin, and atorvastatin. Medication adherence measurement was based on literature review [17, 18] and selected medication possession ratio (MPR) as the measurement. The prescription date of patients who had their first statin medication was treated as the index-date. We followed these patients to trace the occurrences of all-cause or coronary artery disease hospitalizations. Coronary artery disease was based on ICD9-CM codes including acute myocardial infarction (410.90), old myocardial infarction (412), angina pectoris (413.9), coronary artery disease (414.0), and ischemic heart disease (414.9). We also followed up these patients for a three years' follow-up period if there was no hospitalization. These days were the tracking days. The MPR can be presented as follows:

(1) $$\begin{array}{c} \left(\frac{\text{Total days of statin prescription}}{\text{Total tracking days}}\right)\times 100. \end{array}$$

We identified a good medication adherence group (MPR ≧ 80%) and a poor medication adherence group (MPR < 80%) to conduct statistical analyses [19]. All statistical operations were performed using STATA 12 (College Station, Texas, USA).

## 3. Results

There were 19,371 individuals in the final sample of analyses. 59.2% of the sample had MPR < 80%, and 40.8% had MPR ≥ 80%. The average MPR was 63.2 ± 31.97% for all study samples, 40.92 ± 22.02% for the group of MPR < 80%, and 95.54 ± 6.00% for the group of MPR ≥ 80% (Table 1). More women (>53%) than men were in the study population. The average age was 63.14 ± 10.12 years for all study samples. Both groups of MPR < 80% and MPR ≥ 80% had similar average age. Age over 65 had the largest percentage among study samples. For the Charlson Comorbidity Index (CCI), more samples had CCI = 1 compared to CCI = 0 and CCI ≥ 2. For the most visited hospital types, medical centers had the largest percentage, then regional hospitals, district hospitals, and clinics. More samples were seeking care in teaching hospitals (>59%) than in nonteaching hospitals. Samples were also seeking care more in the private hospitals. The samples lived more in the urban areas (>40%) than in the suburban and rural areas. Over 40% of the group of MPR < 80% had hospitalization compared to 22% of the group of MPR ≥ 80% during the study period. The group of MPR ≥ 80% had higher mean of total hospitalization expenditures than the group of MPR < 80%. A small percentage of the samples had emergency visits during the study period. All of these variables were statistically significant (*P* < 0.001) between the group of MPR < 80% and MPR ≥ 80%, except for the age and emergency visits.

Table 2 presents the major variables that affect the probability of having good statin adherence (MPR ≥ 80%). Men had higher probability of having good statin adherence than women (*P* < 0.001). Elderly people had a higher probability of having good statin adherence compared to younger people. People with higher score of Charlson Comorbidity Index had lower probability of having good statin adherence (*P* < 0.05). Compared to seeking care mostly in medical centers, people seeking care mostly in other hospital types had lower probability of having good statin adherence (*P* < 0.001). People seeking care mostly in teaching hospitals had a higher probability of having a good statin adherence compared to people seeking care mostly in nonteaching hospitals (*P* < 0.001). People living in suburban (*P* < 0.001) and rural areas had higher probability of having good statin adherence. People seeking care mostly in private hospitals had lower probability (*P* < 0.001) and those seeking care in nonprofit hospitals had a higher probability (*P* < 0.05) of having good statin adherence compared to people seeking care mostly in the public hospitals.

Table 3 indicates the probability of all-cause hospitalization for study population. The group of MPR ≥ 80% presented significantly lower probability of all-cause hospitalization than the group of MPR < 80% did (*P* < 0.001). Men had a higher probability of all-cause hospitalization compared to women (*P* < 0.001). Compared to age of 45–54, increasing in age also increased the probability of all-cause hospitalization (OR: 1.28 for age of 55–64; OR: 2.34 for age of ≥ 65). Compared to people with CCI = 0, those with higher CCI scores had significantly higher probability of all-cause hospitalization (OR: 1.33 for CCI = 1; OR: 2.42 for CCI ≥ 2). Compared to people seeking care mostly in medical centers, those who seek care mostly in other hospital types had lower probability of all-cause hospitalization. People seeking care mostly in teaching hospitals had a higher probability of all-cause hospitalization compared to people seeking care mostly in nonteaching hospitals. People living in the suburban or rural areas had higher probability of all-cause hospitalization compared to people living in urban areas. People seeking care mostly in private hospitals or nonprofit hospitals had higher probability of all-cause hospitalization compared to people seeking care mostly in public hospitals. However, there is no statistical significance for the hospital ownership.

Table 4 presents the probability of coronary artery disease (CAD) hospitalization. People with MPR ≥ 80% presented lower probability of CAD hospitalization compared to people with MPR < 80% (without statistical significance). Men had higher probability of CAD hospitalization than women did (*P* < 0.001). Compared to age of 45–54, increasing age also increases the probability of CAD hospitalization (OR: 1.40 for age of 55–64; OR: 2.47 for age of ≥ 65). Compared to people with CCI = 0, those with CCI ≥ 2 had significantly higher probability of CAD hospitalization (OR: 1.67) (*P* < 0.001). Compared to people seeking care mostly in medical centers, people seeking care mostly in clinics had lower probability of CAD hospitalization (*P* < 0.001). People seeking care mostly in teaching hospitals had a higher probability of CAD hospitalization compared to people seeking care mostly in nonteaching hospitals (*P* < 0.001). The urbanization type of where people live and hospital ownership did not significantly affect the probability of CAD hospitalization.

Table 5 indicates the probability of emergency visits. Nearly all variables were not significantly affecting the probability of emergency visits, except for people living in rural areas who had a significantly higher probability of emergency visits (OR: 4.58) compared to those living in urban areas (*P* < 0.05).

Table 6 presents the major factors that affect total hospitalization expenditures. Hospitalized patients spent on average NT$177,188 more than those not hospitalized (US$1 = NT$30). People of the group MPR ≥ 80% spent less hospitalization expenditures compared to people of the MPR < 80% group (*P* < 0.001). Men spent more in hospitalization expenditures than women (*P* < 0.001). Increasing age also increases hospitalization expenditures (*P* < 0.001). People with higher CCI scores presented higher hospitalization expenditures (*P* < 0.001). People seeking care mostly in other hospital types spent less in hospitalization expenditures compared to people seeking care mostly in medical centers (*P* < 0.001). People seeking care mostly in teaching hospitals spent more hospitalization expenditures compared to people seeking care mostly in nonteaching hospitals (*P* < 0.001). People living in suburban (*P* < 0.001) or rural (*P* < 0.05) areas spent more in hospitalization expenditures than people living in urban areas. Hospital ownership did not have significant influence on hospitalization expenditures.

## 4. Discussions

Our study results indicated that higher medication adherence will lead to better health care outcomes. These findings are consistent with previous studies [1–3]. Patients who had 80% to 100% medication adherence were significantly less likely to be hospitalized compared with patients with lower levels of adherence. Such adherence-based savings in medical costs are driven primarily by reductions in hospitalization rates at higher levels of medication adherence [3]. Our study results indicated that, for people with MPR ≥ 80%, the probability of all-cause hospitalization is significantly lower than patients of the MPR < 80% group.

Men, with increased age and higher CCI scores, had a higher probability of all-cause hospitalization than women, younger adults, and people with lower CCI scores did. All of these factors could be due to worse health status among men and older adults. Studies also reported lower medication adherence in the older age group [20, 21], and comorbidity was a significant predictor of medication utilization and cost [3]. People seeking care mostly in medical centers or teaching hospitals had the highest probability of all-cause hospitalization compared to people seeking care mostly in other hospital types or nonteaching hospitals. Those people seeking care mostly in medical centers or teaching hospitals have more complicated diseases in general and, thus, increased risk of worse outcomes during the treatment process and may require hospitalizations. People living in suburban or rural areas had higher probability of hospitalization compared to people living in urban areas. In urban areas, health care resources (e.g., physician or hospital bed per 10,000 people) are richer than in suburban or rural areas. People may have better access to health care and receive better care in the urban areas, thus reducing the probability of bad outcomes. Our study also shows that regional barriers to accessing the health care providers may have considerable negative effects on medication adherence. Medication adherence is likely to decrease when patients have difficulties in visiting regularly the health care provider to get medication [11]. Those people with lower medication adherence may increase the probability of bad outcomes. For the probability of coronary artery disease hospitalization, the significant variables are similar to those influencing all-cause hospitalizations. The significances are almost the same, except for hospital types and urbanization.

There was no significant influence for medication adherence level on the probability of emergency visits. This could be due to the problem in Taiwan that many people tend to use more emergency care even though it is not necessary. There are no restrictions of using emergency care in Taiwan. Thus, people are free to choose any emergency care, based on their preferences. Therefore, the influences of medication adherence on emergency care visits are limited.

For total hospitalization expenditures, people with good medication adherence (MPR ≥ 80% group) had significantly less expenditures than people with poor medication adherence (MPR < 80% group). This provides evidence that good medication adherence indeed can reduce medical costs. Those with increased age and higher CCI scores also significantly had higher hospitalization expenditures. This again could be due to the worse health status of these people. People seeking care mostly in medical centers or teaching hospitals had significantly higher hospitalization expenditures than people seeking care mostly in other hospital types or nonteaching hospitals. In general, medical centers or teaching hospitals have more complicated cases for treatment and thus increase the related expenditures. People living in suburban or rural areas also had significantly higher hospitalization expenditures than people living in urban areas. This could be due to worse access to health care that may increase the medical costs.

Medication possession ratio (MPR) was used as the measure of medication adherence in our study. This is based on the recommendations of the International Society for Pharmacoeconomics and Outcomes Research [22]. A systematic review of the methods currently being used to assess adherence and persistence in pharmacoepidemiological and pharmacoeconomic studies indicated that MPR is a popular measurement [23]. Advantages of using MPR measure include the ease of calculation and interpretability [23].

Adherence is a multidimensional phenomenon determined by the interplay of five sets of factors such as social and economic factors, health care team and system-related factors, condition-related factors, therapy-related factors, and patient-related factors [24]. When exploring medication adherence in Taiwan, we consider the impacts from these five factors. First, for social and economic factors, Taiwan introduced universal health care coverage since 1995. Over 99% of population are under coverage. Prescription expenditures are also covered. Therefore, there is good access and limited social barriers to health care and medication. Second, for health care team and system-related factors, under the universal health insurance program, the health care system provides adequate resources to take care of patients having chronic diseases. The medication distribution system is also effective. Third, for condition-related factors, the health status of patients affects illness-related demands. We measured comorbidities of patients by applying Charlson Comorbidity Index (CCI) scores. Thus, such factors have been controlled. Fourth, for therapy-related factors, statins are popularly used worldwide. Statin had become the first choice medication to control lipid and prevent cardiovascular disease [14]. Therefore, its therapy value is recognized in clinical treatment. Fifth, for patient-related factors, perceptions, beliefs, and attitudes of patients will affect their medication adherence behaviors. Nevertheless, it is unlikely to collect this information from claim data. Further researches through questionnaire or interviews may provide better understandings of these issues.

There are two limitations in this study. First, we did not have information on whether people really take the medication or not, despite their medication adherence rates evaluated through MPR. However, other studies indicated that this problem is not unique to our work and may apply to the vast majority of studies, including randomized controlled trials [8]. Second, there is no information regarding the interactions between physicians and patients. Physician's suggestions usually affect the patient's behavior. If a physician spends more time to explain the positive outcomes of good medication adherence and encourages patients to do so, patient's medication adherence may become better. However, no such information exists in the administration databases.

## 5. Conclusion

Good medication adherence brings better outcomes and saves on medical costs for patients who took statin medication. How to motivate patients to keep good medication adherence becomes an important issue in the process of clinical treatment. Effective interventions may be applied to the group of poor medication adherence in order to improve health care outcomes. Further studies on continuously exploring these issues are in great need.

## Figures and Tables

**Table 1:** Characteristics of statin therapy population.

Variable	Study population (%) (*N* = 19371)	MPR < 80 (%) (*N* = 11462)	MPR ≥ 80 (%) (*N* = 7909)	*P* value
Medication possession ratio (MPR) (Mean ± SD)	63.22 ± 31.97	40.92 ± 22.02	95.54 ± 6.00	
Gender				
Women	10825 (55.88)	6610 (57.67)	4215 (53.29)	<0.001^*∗∗∗*^
Men	8546 (44.12)	4852 (42.33)	3694 (46.71)	
Age (mean ± SD)	63.14 ± 10.12	63.04 ± 10.14	63.29 ± 10.10	
45–54	4999 (25.81)	3021 (26.36)	1978 (25.01)	0.100
55–64	6028 (31.12)	3552 (30.99)	2476 (31.31)	
≥65	8344 (43.07)	4889 (42.65)	3455 (43.68)	
Charlson Comorbidity Index (CCI)				
0	7363 (38.01)	4366 (38.09)	2997 (37.89)	<0.001^*∗∗∗*^
1	7866 (40.61)	4511 (39.36)	3355 (42.42)	
≥2	4142 (21.38)	2585 (22.55)	1557 (19.69)	
Hospital type				
Medical center	6718 (34.68)	3546 (30.94)	3172 (40.11)	<0.001^*∗∗∗*^
Regional hospital	5877 (30.34)	3419 (29.83)	2458 (31.08)	
District hospital	3616 (18.67)	2374 (20.71)	1241 (15.70)	
Clinic	3160 (16.31)	2123 (18.52)	1037 (13.11)	
Teaching status				
Nonteaching hospital	6911 (35.68)	4595 (40.09)	2316 (29.28)	<0.001^*∗∗∗*^
Teaching hospital	12460 (64.32)	6867 (59.91)	5593 (70.72)	
Urbanization				
Urban	8411 (43.42)	4933 (43.04)	3478 (43.98)	<0.001^*∗∗∗*^
Suburban	5812 (30.00)	3342 (29.16)	2470 (31.23)	
Rural	5148 (26.58)	3187 (27.80)	1961 (24.79)	
Hospital ownership				
Public hospitals	4954 (25.57)	2835 (24.73)	2119 (26.79)	<0.001^*∗∗∗*^
Private hospitals	8288 (42.79)	5369 (46.84)	2919 (36.91)	
Nonprofit hospitals	6129 (31.64)	3258 (28.42)	2871 (36.30)	
Hospitalization				
No	12634 (65.22)	6481 (56.54)	6153 (77.80)	<0.001^*∗∗∗*^
Yes	6737 (34.78)	4981 (43.46)	1756 (22.20)	
Total hospitalizations expenditures (mean)	130905.90	123689.80	151374.80	<0.001^*∗∗∗*^
Emergency visits				
No	19262 (99.44)	11408 (99.53)	7854 (99.30)	0.084
Yes	109 (0.56)	54 (0.47)	55 (0.70)	

^*∗*^ *P* < 0.05; ^*∗∗*^ *P* < 0.01; ^*∗∗∗*^ *P* < 0.001.

**Table 2:** Multivariate logistic regression model for good statin adherence (MPR ≥ 80%).

Variable	Odds ratio	95% CI	*P* value
Gender			
Women	1		
Men	1.20	1.12–1.29	<0.001^*∗∗∗*^
Age (mean ± SD)			
45–54	1		
55–64	1.07	0.98–1.18	0.138
≥65	1.31	120–1.43	<0.001^*∗∗∗*^
Charlson Comorbidity Index (CCI)			
0	1		
1	1.06	0.98–1.15	0.154
≥2	0.87	0.78–0.97	0.012^*∗*^
Hospital type			
Medical center	1		
Regional hospital	0.74	0.67–0.82	<0.001^*∗∗∗*^
District hospital	0.81	0.72–0.91	<0.001^*∗∗∗*^
Clinic	0.74	0.64–0.84	<0.001^*∗∗∗*^
Teaching status			
Nonteaching hospital	1		
Teaching hospital	1.42	1.26–1.60	<0.001^*∗∗∗*^
Urbanization			
Urban	1		
Suburban	1.28	1.17–1.40	<0.001^*∗∗∗*^
Rural	1.01	0.92–1.12	0.792
Hospital ownership			
Public hospitals	1		
Private hospitals	0.80	0.72–0.87	<0.001^*∗∗∗*^
Nonprofit hospitals	1.12	1.01–1.24	0.029^*∗*^

(1) ^*∗*^ *P* < 0.05; ^*∗∗*^ *P* < 0.01; ^*∗∗∗*^ *P* < 0.001.

(2) Statin adherence (MPR ≥ 80 = 1, MPR < 80 = 0).

**Table 3:** Multivariate logistic regression model for all-cause hospitalization.

Variable	Odds ratio	95% CI	*P* value
Medication possession ratio (MPR)			
MPR < 80%	1		
MPR ≥ 80%	0.32	0.30–0.35	<0.001^*∗∗∗*^
Gender			
Women	1		
Men	1.27	1.19–1.35	<0.001^*∗∗∗*^
Age (mean ± SD)			
45–54	1		
55–64	1.28	1.17–1.40	<0.001^*∗∗∗*^
≥65	2.34	2.15–2.54	<0.001^*∗∗∗*^
Charlson Comorbidity Index (CCI)			
0	1		
1	1.33	1.23–1.43	<0.001^*∗∗∗*^
≥2	2.42	2.22–2.64	<0.001^*∗∗∗*^
Hospital type			
Medical center	1		
Regional hospital	0.96	0.88–1.04	0.285
District hospital	0.87	0.88–1.04	0.011^*∗*^
Clinic	0.62	0.78–0.97	<0.001^*∗∗∗*^
Teaching status			
Nonteaching hospital	1		
Teaching hospital	1.32	1.18–1.47	<0.001^*∗∗∗*^
Urbanization			
Urban	1		
Suburban	1.09	1.01–1018	0.035^*∗*^
Rural	1.24	1.14–1.35	<0.001^*∗∗∗*^
Hospital ownership			
Public hospitals	1		
Private hospitals	1.02	0.94–1.12	0.553
Nonprofit hospitals	1.05	0.96–1.15	0.249

^*∗*^ *P* < 0.05; ^*∗∗*^ *P* < 0.01; ^*∗∗∗*^ *P* < 0.001.

**Table 4:** Multivariate logistic regression model for coronary artery disease hospitalization.

Variable	Odds ratio	95% CI	*P* value
Medication possession ratio (MPR)			
MPR < 80%	1		
MPR ≥ 80%	0.93	0.85–1.03	0.16
Year			
2002	1		
2003	1.00	0.87–1.16	0.98
2004	1.00	0.87–1.15	0.95
2005	0.89	0.77–1.03	0.13
Gender			
Women	1		
Men	1.70	1.54–1.88	<0.001^*∗∗∗*^
Age (mean ± SD)			
45–54	1		
55–64	1.40	1.20–1.64	<0.001^*∗∗∗*^
≥65	2.47	2.15–2.84	<0.001^*∗∗∗*^
Charlson Comorbidity Index (CCI)			
0	1		
1	1.06	0.94–1.20	0.33
≥2	1.67	1.47–1.89	<0.001^*∗∗∗*^
Hospital type			
Medical center	1		
Regional hospital	0.93	0.82–1.04	0.21
District hospital	0.91	0.77–1.08	0.26
Clinic	0.53	0.41–0.69	<0.001^*∗∗∗*^
Teaching status			
Nonteaching hospital	1		
Teaching hospital	1.82	1.52–2.18	<0.001^*∗∗∗*^

^*∗*^ *P* < 0.05; ^*∗∗*^ *P* < 0.01; ^*∗∗∗*^ *P* < 0.001.

**Table 5:** Multivariate logistic regression models for emergency visits.

Variable	Odds ratio	95% CI	*P* value
Medication possession ratio (MPR)			
MPR < 80%	1		
MPR ≥ 80%	1.32	0.28–2.08	0.59
Gender			
Women	1		
Men	1.40	0.52–3.79	0.50
Age (mean ± SD)			
45–54	1		
55–64	1.60	0.40–6.48	0.51
≥65	1.48	0.37–5.83	0.58
Charlson Comorbidity Index (CCI)			
0	1		
1	0.63	0.19–2.03	0.44
≥2	1.18	0.33–4.14	0.80
Hospital type			
Medical center	1		0.06
Regional hospital	0.13	0.02–1.13	0.90
District hospital	0.91	0.21–3.40	0.16
Clinic	0.17	0.01–2.03	0.82
Teaching status			
Nonteaching hospital	1		
Teaching hospital	0.85	0.21–3.47	
Urbanization			
Urban	1		
Suburban	1.03	0.18–5.99	0.98
Rural	4.58	1.13–18.58	0.03^*∗*^
Hospital ownership			
Public hospitals	1		
Private hospitals	0.47	0.13–1.72	0.26
Nonprofit hospitals	0.58	0.14–2.35	0.44

^*∗*^ *P* < 0.05; ^*∗∗*^ *P* < 0.01; ^*∗∗∗*^ *P* < 0.001.

**Table 6:** Multivariate linear regression analysis for total hospitalization expenditures.

Variable	Coefficient	95% CI	*P* value
Hospitalization			
No	1		
Yes	177188.80	175774.2–178603.3	<0.001^*∗∗∗*^
Medication possession ratio (MPR)			
MPR < 80%	1		
MPR ≥ 80%	−16247.12	−17174.97–−15319.26	<0.001^*∗∗∗*^
Gender			
Women	1		
Men	7086.21	6162.18–8010.24	<0.001^*∗∗∗*^
Age (mean ± SD)			
45–54	1		
55–64	2288.41	1011.50–3565.33	<0.001^*∗∗∗*^
≥65	11650.73	10452.76–12848.7	<0.001^*∗∗∗*^
Charlson Comorbidity Index (CCI)			
0	1		
1	7037.78	5941.98–8133.58	<0.001^*∗∗∗*^
≥2	34563.57	33333.87–35793.27	<0.001^*∗∗∗*^
Hospital type			
Medical center	1		
Regional hospital	−2409.216	−3566.50–−1251.93	<0.001^*∗∗∗*^
District hospital	−8282.905	−9983.02–−6582.79	<0.001^*∗∗∗*^
Clinic	−12385.71	−14460.53–−10310.88	<0.001^*∗∗∗*^
Teaching status			
Nonteaching hospital	1		
Teaching hospital	4936.11	3201.40–6670.81	<0.001^*∗∗∗*^
Urbanization			
Urban	1		
Suburban	2792.34	1625.67–3959.02	<0.001^*∗∗∗*^
Rural	1320.59	96.34–2544.83	0.03^*∗*^
Hospital ownership			
Public hospitals	1		
Private hospitals	4691.49	3478.72–5904.27	0.80
NonProfit hospitals	163.94	−1084.44–1412.31	0.93

(1) ^*∗*^ *P* < 0.05; ^*∗∗*^ *P* < 0.01; ^*∗∗∗*^ *P* < 0.001.

(2) US$1 = NT$30.
